# Implementation climate and clinic personnel attitudes in primary care towards a mental health and drug use preventive intervention for hispanic families: preliminary findings

**DOI:** 10.3389/frhs.2025.1619869

**Published:** 2025-09-10

**Authors:** Alyssa Lozano, Elliott R. Weinstein, Tae Kyoung Lee, Justin D. Smith, C. Hendricks Brown, Guillermo Prado

**Affiliations:** ^1^ School of Nursing and Health Studies, University of Miami, Coral Gables, FL, United States; ^2^ Department of Psychiatry, Massachusetts General Hospital, Boston, MA, United States; ^3^ Department of Child Psychology and Education/Social Innovation Convergence Program, Sungkyunkwan University, Seoul, Republic of Korea; ^4^ Department of Population Health Sciences, Spencer Fox Eccles School of Medicine at the University of Utah, Salt Lake City, UT, United States; ^5^ Department of Psychiatry and Behavioral Sciences, Feinberg School of Medicine, Northwestern University, Chicago, IL, United States

**Keywords:** implementation climate, evidence-based practices, mental health, drug use, primary care

## Abstract

Despite the availability of preventive interventions to address mental health and drug use among Hispanic adolescents, few are implemented in real-world settings. Favorable attitudes towards evidence-based practices and a better implementation climate can facilitate the successful execution of interventions in real-word settings to ameliorate health disparities among Hispanic youth. The purpose of this study was to investigate how implementation climate influences attitudes toward a mental health and drug use preventive intervention for Hispanic families at the individual and clinic level. Participants included 73 clinic personnel from 18 primary care clinics that were part of an effectiveness-implementation hybrid Type 1 study in South Florida. Clinic personnel completed the Implementation Climate Scale and Evidence-based Practice Attitude Scale. Using hierarchal linear modeling, we examined: (1) whether individual differences in implementation climate were associated with individual attitudes towards an evidence-based practice within clinics, and (2) whether clinic-level differences in mean implementation climate were associated with clinic-level differences in attitudes towards an evidence-based practice. At the individual level, there was a significant positive relationship between individual implementation climate and attitudes toward the evidence-based practice. Implementation climate varied significantly among individuals. At the clinic level, clinics with higher average implementation climate did not show significantly different average attitudes towards the mental health and drug use preventive intervention. Understanding implementation climate and attitudes toward evidence-based practices can inform tailored implementation strategies for the unique needs of primary care settings to address drug use and mental health disparities among Hispanic youth.

## 1 Introduction

Mental health problems, including depression, anxiety, suicidal thoughts and behaviors, and drug use are increasingly prevalent across adolescence. This is especially true for Hispanic youth who often experience several of these challenges concurrently. For example, compared to the general population of United States (U.S.) high school students, Hispanic high school students reported higher rates of suicidal behaviors in the past year including having serious suicidal thoughts (16.7% vs. 15.8%) and suicide attempts (10.2% vs. 7.8%) (1). Additionally, among Hispanic adolescents who reported lifetime and past 3-month illicit drug use, 20.8% also reported suicide ideation and 12.5% reported suicide attempts, compared to 8.4% and 5.2% of adolescents who did not report lifetime and past 3 month illicit drug use, respectively (2).

Culturally syntonic preventive interventions are needed to help ameliorate behavioral health disparities among Hispanic youth. Although interventions to address multiple behavioral health outcomes among this underserved population exist, most have only been evaluated in research settings, creating uncertainty as to how well they may delivered or work in real-world settings (3). Evaluating evidence-based, preventive interventions in real world settings through hybrid effectiveness-implementation studies (4), is critical to understanding whether an intervention can be successfully implemented into specific settings, ensuring future widespread dissemination. Inclusive dissemination can broaden an intervention's reach so that populations in need can access concrete resources to help eliminate individual, family, and systemic risk factors for behavioral disorders and drug use (5).

Considering the importance of widely disseminating preventive interventions for Hispanic youth, it is imperative to identify settings that may be conducive to their implementation and future dissemination and scale-up efforts; one such setting being primary care. Pediatric primary care settings can support integrated care models that include preventive interventions as the standard of care (6–8), thereby creating alternative options for the implementation of evidence-based interventions. The implementation of evidence-based interventions for Hispanic youth is particularly important as mental health services are vastly underused among Hispanic populations (9). Largely due to stigma around seeking care, Hispanic populations are less likely to access mental health services compared to non-Hispanic white populations and tend to rely more on informal support networks (10–13). However, Hispanic families often attend primary care clinics for annual visits, and physicians are trusted sources of information (14, 50); and as such, may be more amenable to participating in preventive interventions if it is recommended by someone they know, such as a primary care physician (51, 52).

Despite the promise of primary care as a vehicle for the successful implementation and dissemination of preventive interventions for Hispanic youth, challenges remain in ensuring the successful implementation/dissemination of evidence-based preventive interventions (15). Across primary care clinics, there may be challenges in implementing evidence-based behavioral preventive interventions because of possible disruptions to clinic flow and interference with clinic personnel's standard job requirements (50). Additionally, certain clinics and their personnel may be less receptive to the implementation of evidence-based preventive interventions because they do not perceive that the adoption, implementation, and use of an evidence-based preventive intervention is expected, rewarded, and supported by their respective clinic leadership (16, 17). Such factors comprise a major part of what is known as implementation climate (17). Information regarding the implementation climate of a given setting (e.g., pediatric primary care clinic) can provide researchers, implementation intermediaries, and other implementation supporters (e.g., champions, facilitators) with information about the organizational context of the setting they are working in.

As such, measuring implementation climate is key to effectively implement evidence-based preventive interventions into real-world settings. With specific information about the extent to which an organization prioritizes and values the successful implementation of evidence-based preventive interventions, implementers can identify targets to improve the likelihood of fuller implementation across settings (17). In the context of primary care clinics, implementation climate may vary due to certain characteristics of the clinic as well as personnel within the clinic. These heterogeneous clinics have different resources, structures, and capabilities that may impact clinics' implementation climate (18, 19). Relatedly, across the various types of primary care clinics, clinic personnel may lack time the time and resources to assess the need for, engage with and/or deliver the preventive intervention eHealth Familias Unidas Mental Health and may subsequently perceive a poor implementation climate if they are not supported by their clinic's administration to take on additional tasks (20).

A poor implementation climate may impact personnel's attitudes towards a desired evidence-based practice (i.e., preventive intervention) being implemented within the clinic (21, 22). According to the Consolidated Framework for Implementation Research [CFIR; Damschroder et al. (23, 24)], both organizational factors, such as implementation climate, and individual characteristics, such as attitudes toward evidence-based practices, are key determinants of successful implementation. Understanding these factors (i.e., implementation climate and attitudes towards evidence-based practices) is particularly important in primary care settings serving Hispanic youth, who face persistent and under addressed health disparities. Negative attitudes toward evidence-based programs among clinic personnel can reduce willingness to engage with new interventions, potentially undermining program fidelity, reach, and effectiveness (22). This underscores the importance of understanding how organizational context shapes individual attitudes to inform effective implementation strategies. While CFIR provides a framework for identifying multilevel determinants of implementation success, the Social Development Model (25) can help illuminate the mechanisms through which organizational environments influence individual behavior. By emphasizing that prosocial behaviors emerge when individuals are provided with meaningful opportunities for involvement, supported in skill development, and reinforced for their efforts, a strong implementation climate can foster engagement with EBPs by aligning expectations, building provider capacity, and offering recognition (25). Therefore, identifying how organizational context (i.e., climate) shapes individual attitudes is critical for informing tailored implementation strategies to support implementation and sustainment of behavioral preventive interventions for Hispanic youth.

Although the association between implementation climate and attitudes toward evidence-based practices is well document in the extant literature, the majority of this research has largely been conducted in specialized healthcare settings such as substance use treatment (26) and mental health clinics (27), with less attention paid to primary care clinic settings which can be leveraged to reach Hispanic families. This study addresses this gap by examining how implementation climate influences attitudes toward evidence-based practices (i.e., eHealth Familias Unidas Mental Health) at the individual and clinic level, thus contributing to a deeper understanding of how to integrate preventive interventions into primary care to ameliorate health disparities in mental health and drug use among Hispanic youth.

## 2 Method

### 2.1 Participants and procedures

Participants included 73 clinic personnel who were affiliated with clinics that were part of the eHealth Familias Unidas Mental Health study (53). eHealth Familias Unidas Mental Health study is an effectiveness-implementation hybrid Type 1 study that uses a rollout trial design with 18 primary care clinics in South Florida to evaluate both effectiveness and implementation outcomes related to a wide range of behavioral health issues among Hispanic youth (i.e., drug use and poor mental health; 53). Individual/participant-level effectiveness is determined by whether randomization to eHealth Familias Unidas Mental Health prevents/reduces depressive, anxiety symptoms; suicide ideation and behavior; and drug misuse compared to those randomized to prevention as usual. Concerning implementation, the parent study evaluates whether sustainment of the intervention and intervention impact on study outcomes (i.e., mental health and externalizing behaviors) vary as a function of the quality of implementation at the levels of clinic and clinician.

To evaluate the implementation related outcomes, we asked clinic staff (e.g., nurse assistants, mental health counselors, or any other health professional located within the participating clinics), facilitators, leaders, and physicians, hereafter clinic personnel, various implementation-related factors related to implementation climate and attitudes towards evidence-based practices (see measures below). Participant characteristics, including clinic personnel's specific role, can be found in Table 1. Clinic personnel were compensated $20 for their time in completing the survey which was sent electronically via email using REDCap software (28). Clinic personnel completed informed consent prior to completing the survey. All study procedures were approved by the University of Miami Social and Behavioral Sciences Institutional Review Board.

**Table 1:** Participant characteristics.

Variable	*N* (%) or *M* (SD)
Role in clinic	
Clinical Director	8 (11.6%)
Physician	11 (15.9%)
Office Manager	7 (10.1%)
Nurse	3 (4.3%)
APRN	2 (2.9%)
Mental Health Professional	15 (21.7)
Staff	5 (7.2%)
Other	18 (26.1%)
Study facilitator	
Yes	22 (31.9%)
No	47 (68.1%)
Clinic type	
FQHC	28 (40.6%)
Private Clinic	9 (13.0%)
Community Clinic	12 (17.4%)
Hospital System	6 (8.7%)
Academic Setting	14 (20.3%)
Implementation Climate	43.80 (17.55)
FQHC	46.31 (18.26)
Private Clinic	36.00 (21.45)
Community Clinic	44.03 (10.49)
Hospital System	46.50 (5.54)
Academic Setting	45.00 (19.03)

### 2.2 Measures

#### 2.2.1 Clinic personnel characteristics

We created a variable to classify clinic type. Although all clinics were primary care clinics, they were heterogeneous and consisted of Federally Qualified Health Centers, clinics within academic medical settings, private clinics, community clinics, and hospital system affiliated clinics.

#### 2.2.2 Implementation climate scale

The Implementation Climate Scale (ICS; 18 items; α = .96; 17) was used to assess the degree to which there is a strategic organizational climate supportive of evidence-based practices (EBP, e.g., eHealth Familias Unidas Mental Health) implementation. The ICS captures six dimensions of the organizational context that indicate to employees the extent to which their organization prioritizes and values the successful implementation of EBP: (1) selection for openness, (2) recognition for EBP, (3) selection for EBP, (4) focus on EBP, (5) educational support for EBP, and (6) rewards for EBP. Sample questions included: “One of this team/agency's main goals is to use evidence-based practices effectively” and “This team/agency selects staff who value evidence-based practices”. Response options ranged from 0 (not at all) to 4 (to a very great extent) with higher averages indicating a positive implementation climate.

#### 2.2.3 Evidence-based practice attitude scale

The Evidence-based Practice Attitude Scale (EBPAS; 15 items; α = .92; 29) evaluated clinic personnel's attitudes towards the adoption of evidence-based practices (i.e., eHealth Familias Unidas Mental Health). The constructs of the EBPAS include [1] willingness to adopt EBP given their intuitive appeal, [2] willingness to adopt new practices if required, [3] general openness toward new or innovative practices, and [4] perceived divergence of usual practice with academically developed or research-based practices. Example questions were: “I am willing to try new types of therapy/interventions even if I have to follow a treatment manual” and “If you received training in a therapy or intervention that was new to you, how likely would you be to adopt it if it was intuitively appealing?” Responses ranged from 0 (not at all) to 4 (to a very great extent) with higher averages indicating a positive attitudes toward eHealth Familias Unidas Mental Health.

### 2.3 Data analysis plan

We conducted a two-level modeling approach (i.e., hierarchal linear modeling) to account for the hierarchical nature of the data, where individuals (Level 1) were nested within clinics (Level 2). This approach allowed us to examine [1] whether individual differences in implementation climate were associated with individual attitudes toward eHealth Familias Unidas Mental Health within clinics, and [2] whether clinic-level differences in mean implementation climate predict clinic-level differences in attitudes toward eHealth Familias Unidas Mental Health. We also estimated implementation climate on the “between level” to assess whether clinics varied significantly in their mean implementation climate. Guided by the Social Development Model we also sought to evaluate the association between recognition for EBP, educational support for EBP, and rewards for EBP and attitudes toward eHealth Familias Unidas Mental Health at the within-level. In addition to evaluating each dimension separately, we created a binary index variable representing a minimum score threshold across all three dimensions, such that individuals were coded as meeting the threshold only if they had a score of one on all three. This index was included in the model to assess whether joint perceptions of recognition, educational support, and rewards were associated with more favorable attitudes toward eHealth Familias Unidas Mental Health. To aid in the evaluation of the strength of the effects, we calculated standardized regression coefficients as per Hox, Moerbeek (30). All analysis were performed in M*Plus* 8.0 (31) using full information maximum likelihood to handle missing data. We also examined descriptive statistics to assess whether there were significant differences in implementation climate across clinic types (i.e., Federally Qualified Health Centers, clinics within academic medical settings, private clinics, community clinics, and hospital system affiliated clinics).

## 3 Results

Intraclass correlations (ICCs) of implementation climate and attitudes toward were .025 and .127, respectively. These ICCs suggested that 99.75% of the variance in implementation climate and 87.3% of the variance in attitudes toward was attributable to within-person deviations.

### 3.1 Within-level (individual; level 1)

At the within level, there was a significant positive relationship between individual implementation climate and attitudes toward eHealth Familias Unidas Mental Health. Specifically, a one-standard deviation (SD) increase in implementation climate corresponded to a 0.80 SD increase in attitudes toward eHealth Familias Unidas Mental Health (*β* = .802, SE = .084, *p* < .001). Moreover, implementation climate varied significantly among individuals (variance = 203.933, *p* = .001). When evaluating the three dimensions of the ICS, recognition for EBP (*β* = .362, SE = .175, *p* < .05) was significantly associated with increased attitudes toward eHealth Familias Unidas Mental Health.

### 3.2 Between-level (clinic; level 2)

By contrast, at the between level, clinics with higher average implementation climate did not show significantly different average attitudes toward eHealth Familias Unidas Mental Health (*β* = –0.854, SE = 2.48 *p* = .731). Although the average of implementation climate across clinics was significant and showed marginally significant variance (variance = 29.77, *p* = .056), nearly all unexplained variance in attitudes toward eHealth Familias Unidas Mental Health at the between level was negligible (0.870, *p* = .962). The lack of findings at the between level is consistent with the low ICC.

## 4 Discussion

The purpose of this study was to provide preliminary evidence regarding how implementation climate influences attitudes toward eHealth Familias Unidas Mental Health, a behavioral health intervention for Hispanic youth, at the individual and clinic levels. We found that clinic personnel (individual level) with higher perceptions of their organization's implementation climate tend to have significantly higher attitudes toward eHealth Familias Unidas Mental Health. Moreover, clinic personnel with higher perceptions of recognition for engaging with eHealth Familias Unidas Mental Health also had significantly higher attitudes towards it. At the clinic level, we found that clinics with higher average implementation climate did not systematically show higher (or lower) average attitudes toward eHealth Familias Unidas Mental Health. These preliminary results suggest that individuals' perception of their organization's implementation climate exerts a meaningful influence on attitudes toward evidence-based practices within clinics (i.e., at the individual level) while implementation climate does not appear to systematically predict differences in attitudes toward eHealth Familias Unidas Mental Health between clinics (i.e., at the clinic level).

Within our study, there was a positive association between clinic personnel's perception of their organization's (1) implementation climate in general and (2) recognition for engaging with the EBP and attitudes towards eHealth Familias Unidas Mental Health. This finding is unsurprising given that implementation climate reflects the perceptions that evidence-based practices will be expected, rewarded, and supported within a certain health context (16). Thus, individuals with positive (or higher) perceptions of their organization's implementation climate are likely to have more positive attitudes towards evidence-based practices if they feel like their organization will recognize them for engaging with the evidence-based practice, in this case, eHealth Familias Unidas Mental Health. Recognition for EBP use may serve as a key motivational mechanism that fosters more positive attitudes and sustained engagement among clinic personnel (17). This is particularly important to assess prior to implementation, which is when our survey was administered, to determine what aspect(s) of a clinic's implementation climate leaders should focus on. This finding is consistent with past research, both qualitative and quantitative, suggesting that engaging and supporting clinic personnel is a necessary component in adopting new evidence-based practices, such as eHealth Familias Unidas Mental Health, within primary care settings (50). Although other individual-level variables were not evaluated in this study, personal beliefs about the relevance or cultural fit of the intervention, perceived self-efficacy, and burnout or workload may influence attitudes towards EBPs. Moreover, prior experience with EBPs, individual role (e.g., physician vs. medical assistant), and level of engagement with previous research or training initiatives may also modify the relationship between implementation climate and attitudes towards EBPs such as eHealth Familias Unidas Mental Health. These variables should be evaluated in future research to understand individual-level characteristics could interact with the broader implementation climate to either strengthen or dampen support for EBPs.

No meaningful differences in implementation climate and the association between implementation climate and attitudes toward eHealth Familias Unidas Mental Health across clinics (i.e., between level) emerged. Moreover, when evaluating the mean implementation climate across clinic types (i.e., Federally Qualified Health Centers, clinics within academic medical settings, private clinics, community clinics, and hospital system affiliated clinics), we did not find significant differences. This finding, or lack thereof, may be due to the small number of clinics surveyed (*n* = 18) and their shared geographic context within Miami-Dade County, which may have limited variability and statistical power to detect significant between-clinic effects. Nonetheless, the lack of differences across clinics and clinic types is interesting given that these clinics may vary in their size and available resources, which may subsequently contribute to their capability/willingness towards implementing new evidence-based practices. Some clinics may have more access to resources such as physical space, supports from federal or state government systems, and providers/staff members with more widespread expertise or skills sets that might endorse a positive implementation climate (18, 32). Prior literature suggests that healthcare settings that are better resourced are more willing, and even excited, to execute new evidence-based interventions within their healthcare settings (33, 34).

It is possible that the lack of differences observed across clinics may be due to the fact that irrespective of clinic type and available resources, key organizational factors such as implementation support or clinic culture were consistent across sites. It may be that all clinics have a “clinical champion” or “facilitator” (e.g., chief medical officer, senior physician, owner of clinic) who can inspire staff to see the value of administering new services. This may facilitate a more positive implementation climate (35–38). Clinics may have also had comparable levels of leadership engagement and infrastructure to support EBPs that subsequently contribute to a relatively uniform implementation climate. Additionally, these clinics may have a history of adopting and disseminating academic research interventions; and therefore, may be more interested in implementing future evidence-based practices due to a general ethos of positivity towards disseminating academic research (39).

### 4.1 Implications

The findings of this preliminary study have important implications for the implementation of behavioral health preventive interventions in real world settings. Primary care clinics are often the first, and most frequently visited, medical setting for many children and families, and thus hold remarkable potential for the implementation of behavioral preventive interventions (40, 41). Understanding implementation climate and attitudes toward evidence-based practices prior to implementation can inform implementation strategies and meet the unique needs of personnel within different primary care settings. To increase the potential for success in implementing and disseminating efficacious evidence-based practices, it is crucial to establish collaborative relationships with clinic personnel, from patient-facing staff to senior administrators, to ensure implementation success. When engaging with clinic personnel, research teams should not only focus on the importance of a collaborative relationship between the clinic and research team but also take time to understand clinic characteristics that may impact implementation of the intervention. One possible strategy is to include pre-implementation planning wherein researchers evaluate the implementation climate of a given clinic and its personnel, and based on their those findings, develop a formal implementation blueprint for the given clinic (39, 42, 43). To maximize impact, specific strategies within the developed blueprint should be developed collaboratively with the research team and clinic personnel (44).

Another example of a possible implementation strategy to improve clinic-level climate includes education-oriented strategies which consist of activities that encourage adoption and high-quality delivery of evidence-based practices, based on the perceived benefits of the program for the clinic's patients [i.e., education-oriented strategies (45)]. Intervening on clinic personnel alone will have little effect on the overall climate if clinic leaders do not alter their expectations, recognition, and incentives for adopting and implementing new EBPs; thus, education-oriented strategies should be focused on both clinic personnel *and* clinic leadership as those in leadership will ultimately determine priorities, set incentives and evaluation criteria/metrics related to the EBP that may impact how clinic personnel perceive the clinics implementation climate (46–48). As highlighted in our findings, if clinic personnel feel as though they will be recognized for engaging with the EBP (e.g., seen as clinical experts, held in high esteem), they may be more willing to adopt and implement new EBPs. Intervening at the clinic level may subsequently improve clinic personnel's openness to and focus on the EBP if leaders promote and support use of the EBP.

Intervening at the clinic-level underscores the importance of tailoring implementation strategies to not only individual attitudes, but also to the broader clinic context (45). Among clinics, strategies such as engaging clinic leadership to champion preventive efforts, incorporating training on culturally responsive care, and streamlining referral and follow-up processes can enhance adoption and long-term sustainability of evidence-based preventive interventions for vulnerable populations. Strategies such as embedding preventive programs into existing clinic workflows (e.g., during well-child visits) and using clinical decision support tools can also help normalize and routinize delivery of evidence-based practices (49), thereby strengthening the overall implementation climate. Improved personnel perception of their clinic's implementation climate may then improve attitudes toward the specific intervention. Although not evaluated in this study, these factors may then impact intervention attendance and actual intervention outcomes for youth. As noted, recognition for engagement with eHealth Familias Unidas Mental Health was associated with positive attitudes towards the intervention. Thus, strategies such as offering financial incentives (e.g., salary increases, bonuses), organizing professional development and educational opportunities, and even decreasing day-to-day responsibilities for clinic personnel who are involved with the intervention may not only help to increase the likelihood of implementer buy in, but also bolster attitudes towards EBPs more broadly within these primary care settings.

Irrespective of an individual's perception of implementation climate, research teams should ensure that these personnel have the necessary supports to be fully invested in the delivery and success of the intervention. In our own study, although the research team works to engage and maintain contact with the primary care clinics and involves clinic personnel in study decision making related to study processes and procedures, it may be that there is a need to spend more time problem solving with clinics to improve implementation climate. For example, focusing on tracking clinic leaders' progress in established leadership development plans pertaining to the EBP, updating the plans based on emergent issues or needs, providing additional leadership support, and identifying organizational needs may be tailored and adaptive approaches to align organizational-level initiatives with clinic-level activities to achieve positive changes in implementation leadership and climate (46, 47).

Overall, research teams need to be mindful of the role of implementation climate in selection and inclusion of practice sites for implementation (and effectiveness) research. Although it might seem reasonable and even advantageous to engage clinics in research that have a more positive climate for implementing new EBPs, doing so invariably means excluding practice sites and individuals that are arguably in greater need of practice change and innovation. This could result in the perpetuation of disparities in EBP access for certain patient populations and the widening of health inequities. To avoid further these disparities, research teams ought to consider implementation preparation activities prior to starting implementation of new EBPs that focus on improving implementation climate and other organizational factors to level the field so to speak across diverse clinics. This is a feature of an equitable implementation approach to translation to underserved communities and populations that experience disparities (54).

### 4.2 Limitations and strengths

The present study findings should be interpreted while considering the following limitations. First, this preliminary study was cross sectional and did not capture how both implementation climate and attitudes toward evidence-based practices may change across time as clinic personnel are more exposed to eHealth Familias Unidas Mental Health and thus have improved attitudes toward the intervention. Second, we did not collect demographic information from the clinic personnel which limits our ability to understand how sociodemographic characteristics may impact both implementation climate and attitudes towards eHealth Familias Unidas Mental Health. Future studies should collect sociodemographic and contextual data (i.e., staff turnover, clinic size, prior experience with EBPs) on clinic personnel and clinics, respectively, to further ground findings. Third, all clinics were in South Florida, limiting the generalizability of the findings to other areas. Finally, all data were collected via self-report, which introduces the possibility of response bias, including social desirability and inaccurate recall. This may affect the accuracy of reported attitudes and perceptions.

Despite these limitations, this study evaluated how implementation climate influences attitudes toward a mental health and drug use preventive intervention for Hispanic families. Findings from this study can provide insight related to factors that may facilitate or hinder the process of implementing behavioral preventive interventions in primary care settings. A better understanding of such factors can lead to the development of tailored implementation strategies that can meet the unique needs of different primary care settings and ensure successful implementation of preventive interventions to prevent and reduce mental health and drug use outcomes among Hispanic youth.

## Data Availability

The raw data supporting the conclusions of this article will be made available by the authors, without undue reservation.
